# Models in the Research Process of Psoriasis

**DOI:** 10.3390/ijms18122514

**Published:** 2017-11-24

**Authors:** Katarzyna Bocheńska, Elwira Smolińska, Marta Moskot, Joanna Jakóbkiewicz-Banecka, Magdalena Gabig-Cimińska

**Affiliations:** 1 Department of Medical Biology and Genetics, University of Gdańsk, Wita Stwosza 59, 80-308 Gdańsk, Poland; katarzyna.bochenska@phdstud.ug.edu.pl (K.B.); elwira.smolinska@biol.ug.edu.pl (E.S.); marta.moskot@biol.ug.edu.pl (M.M.); joanna.jakobkiewicz-banecka@biol.ug.edu.pl (J.J.-B.); 2 Department of Physiology, Medical University of Gdańsk, Dębinki 1, 80-211 Gdańsk, Poland; 3 Institute of Biochemistry and Biophysics, Polish Academy of Sciences, Laboratory of Molecular Biology, Kładki 24, 80-822 Gdańsk, Poland

**Keywords:** psoriasis, disease models, pathomechanism, biomedical tools, drug-development

## Abstract

Psoriasis is an ancient, universal chronic skin disease with a significant geographical variability, with the lowest incidence rate at the equator, increasing towards the poles. Insights into the mechanisms responsible for psoriasis have generated an increasing number of druggable targets and molecular drugs. The development of relevant in vitro and in vivo models of psoriasis is now a priority and an important step towards its cure. In this review, we summarize the current cellular and animal systems suited to the study of psoriasis. We discuss the strengths and limitations of the various models and the lessons learned. We conclude that, so far, there is no one model that can meet all of the research needs. Therefore, the choice model system will depend on the questions being addressed.

## 1. Psoriasis and Research of This Disease

Psoriasis (Ps) is a chronic inflammatory skin disease affecting about 2–3% of the worldwide population. Ps is characterized by infiltration of immune cells, epidermal hyperproliferation, and abnormal keratinocyte differentiation [1]. Many studies have reported various factors contributing to the pathogenesis of Ps, including genetic factors, the immune system, and environmental conditions, thus recognizing it as a multifactorial disease [2,3].

**Figure 1:**
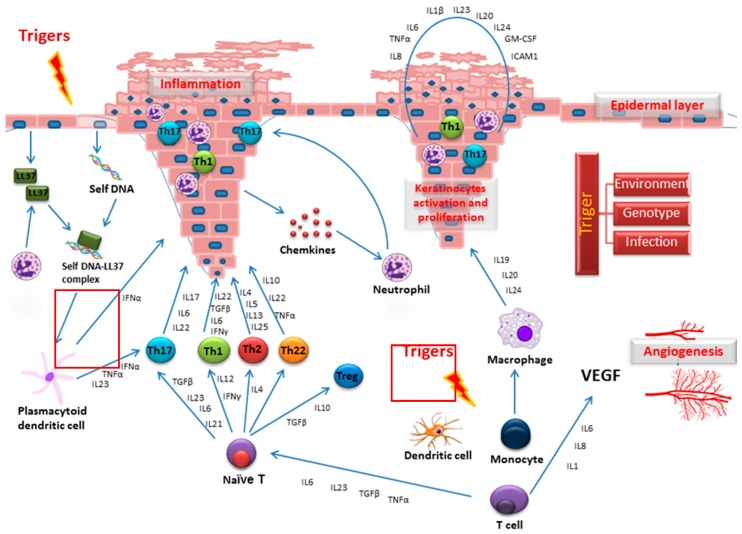
Key signals in psoriasis (Ps) development.

There are five types of Ps: plaque, guttate, inverse, pustular, and erythrodermic. The most common form, plaque Ps, typically has raised red or white scaly skin lesions with a thickened acanthotic epidermis. Key findings in the affected skin of patients with Ps include vascular engorgement due to superficial blood vessel dilation and altered epidermal cell cycle. Such changes are thought to be related to the various inflammatory cytokines released in the inflammatory process, such as tumor necrosis factor-α (TNF-α), interferon-γ (IFN-γ), and interleukin-17 (IL-17). Altered differentiation of psoriatic keratinocytes is characterized by an upregulation of the early differentiation markers (involucrin, small proline-rich proteins, keratin 6, keratin 16, and keratin 17), and downregulation of the late keratinocyte differentiation markers (filaggrin, loricrin, and caspase-14). Key signals in Ps development are depicted in Figure 1.

Within the last decade, substantial advances have been made in elucidating the molecular pathogenesis of Ps. Transcriptomic analyses have been widely used to identify differentially expressed genes (DEGs) associated with Ps pathology [4,5,6,7,8,9,10]. Genome-Wide Association Studies (GWAS) have identified multiple genetic variants and Ps susceptibility genes. Although there is now increasing insight into the genes conferring disease susceptibility, much less is known about the regulatory networks of expressed genes that define the molecular signature of the disease. Psoriatic lesional (PP) skin has been shown to have a pattern of gene expression that is different from that of psoriatic noninvolved (PN) skin [6,11]. Also, several transcription factors (TFs) that are activated or inhibited in the Ps transcriptome has been identified. In addition to important mRNA expression alterations, Ps is characterized by a specific microRNA expression profile, distinct from that of healthy skin [12]. Also, a meta-analysis has produced a reference list of consistent candidate genes for further investigation of Ps pathology and new therapeutic targets [9].

Despite our increasing knowledge of Ps, there have been several attempts to reproduce the disease in in vitro and in vivo models to gain more detailed insights into mechanisms of disease pathogenesis, as well as therapeutic strategies. Here, we review the wide range of current Ps models and summarize their advantages, disadvantages, and suitability for illustrating specific aspects of this dermatosis. Some models have been used to study varied conditions of Ps, and new models are still being developed. Some of these models confirmed existing hypotheses, whereas others have provided important and even unexpected new insights into steps of the pathogenic cascade presented in this disease. However, all of these models have increased our knowledge of Ps pathogenesis, as well as provided insights relevant to therapeutic strategies.

To address restrictions on the feasibility of animal models and ethical concerns, in vitro models have been developed. Besides, the need for an improved understanding of the progression and treatment of Ps has pushed for increased accuracy and physiological relevance of in vitro culture systems. As a result, these models have increased in complexity, and their output parameters have further diversified as they have progressed beyond simple proliferation and begun recapitulating critical steps in the inflammatory cascade [13]. Furthermore, tissue engineering has enabled rapid development of new in vitro reconstituted human epidermal culture models to mimic the morphology of the normal stratified squamous epidermis. To date, most cell culture and tissue engineering strategies rely on established cell lines or primary epidermal keratinocytes derived from Ps patients or normal individuals. Limitations of in vitro Ps models include the absence of blood vessels and microenvironments; nevertheless, these models are useful for investigating the molecular mechanism of the disease, including keratinocyte differentiation and response to stimuli [14] (Table 1).

In vivo systems (living organisms) have also been used as Ps models. The use of animals as research models was initiated by Claude Bernard in the 19th century [15], and animal models are now widely used in research. An animal model is considered homologous if the symptoms shown by the animal and the course of the condition are identical to those of humans (this is rare), and is considered isomorphic if the symptoms are similar but the cause of the symptoms differs between human and the model. However, most models are neither homologous nor isomorphic and are instead referred to as partial [16]. Most animal disease models can be categorized as (i) spontaneous, (ii) genetically modified, (iii) induced, (iv) negative, or (v) orphan, of which the first three are most abundant. Spontaneous and transgenic models are genetic variants, which mimic the human condition. Induced models are characterized by creation, either through surgical or genetic modifications, or chemical injections. Negative models are mainly used to study the disease resistance mechanism, while orphan models are animals without a corresponding disease in humans [17].

Ps appears to be restricted to humans and does not occur in animals, except rhesus and cynomolgus monkeys [18]. Also, sporadic cases of Ps-like-phenotypes in dogs and pigs have been reported [19,20]. An ideal Ps model should reflect the clinical hallmarks of human Ps, including characteristic histomorphologic features, and similar pathogenesis, and should respond similarly to therapeutic agents. However, the heterogeneous mechanisms of Ps make it difficult to identify a model that completely mirrors all aspects of the disease [13]. Mouse models have been successfully employed to mimic several aspects of human skin diseases, including Ps, contact hypersensitivity, wound healing, and atopic dermatitis [21]. Species differences between rodents and humans is a major problem associated with in vivo models of Ps. Also, despite many common molecular and immunological pathways and the similar stratification of mouse and human skin, there are multiple important anatomical and cellular differences between these two species (Table 1).

## 2. In Vitro Models

### 2.1. Two-Dimensional (2D) Engineered Skin Psoriatic Cell Model

**Figure 2:**
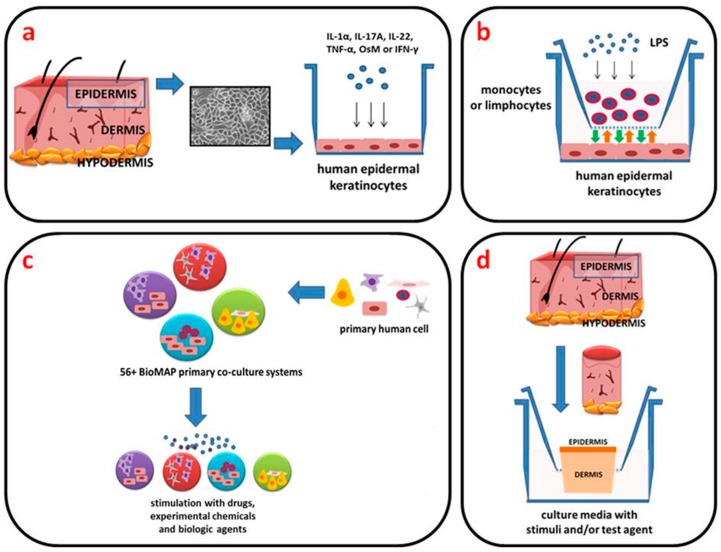
Engineered Ps models. (**a**) The two-dimensional (2D) engineered skin psoriatic cell model; (**b**) The co-culture system; (**c**) The biologically multiplexed activity profiling (BioMAP) system; (**d**) The reconstituted human epidermal model (3D).

The absence of a specific profile that defines the psoriatic phenotype necessitates the use of physiologically relevant and reliable in vitro models to investigate this disorder and develop more effective treatments. Monolayer keratinocyte cultures have been widely used for biological and pharmacological studies and screening of anti-psoriatic drugs because they provide an easily reproducible first step model system [2,11,12,22]. To mimic the clinical situation as closely as possible, an evident source is needed to present keratinocytes derived from psoriatic plaques, although psoriatic keratinocytes are difficult to culture [23,24]. Initial attempts to generate an in vitro model of psoriasis (Ps) were made using skin cells from freshly isolated lesional psoriatic skins. However, authors reported that the disease phenotype, as assessed by examining the expression of four selected Ps-associated genes (*CAMP*, *DEFB4*, *PI3*, and *TNF*), was lost during in vitro cell culture expansion [23]. To maintain the psoriatic phenotype, the addition of cytokines was required. However, considering the lack of reproducibility and the difficulties associated with sourcing isolated lesional psoriatic skins, this approach is inefficient and does not meet the needs of a reliable, accessible, and predictive preclinical model. Therefore, two commonly studied cell lines (normal human epidermal keratinocytes (NHEK) and immortalized human keratinocyte line (HaCaT)) are often used for the study of Ps. In contrast to many virally transformed keratinocyte cell lines, HaCaT is capable of expressing differentiation-specific gene products, including keratins 1 (KRT1) and 10 (KRT10), and differentiation markers such as involucrin and filaggrin [25]. Furthermore, a much broader spectrum of keratins than that usually seen in primary keratinocyte cultures is expressed by HaCaT, including keratins associated with simple epithelia (e.g., KRT7, KRT8, KRT18, and KRT19). When approaching very high cell densities, the expression of the suprabasal keratins increases, while the constitutively synthesized simple keratins (mainly KRT7, KRT8, and KRT19) decrease [25]. As mentioned above, Ps-associated features are induced by controlled addition of selected cytokines in order to mimic disease pathogenesis mechanisms. The most commonly studied cytokines in this context are strongly associated with either innate (IL-1α, IL-6, and TNF-α) or adaptive immune responses (IL-17A). The choice of this specific set of cytokines was made on the basis of information reported by several research groups [6,26,27,28,29,30,31,32,33,34], addressing cytokine stimulation of keratinocytes (Figure 2a). For selection of the best proinflammatory mix to create a Ps model, a detailed evaluation of the individual and possible synergistic effect of selected cytokines (IL-1α, IL-17A, IL-22, oncostatin-M [OsM], TNF-α, and IFN-γ) on the expression of Ps-associated genes was made [35,36]. IL-1α and TNF-α exhibit analogous regulation patterns, particularly with respect to the expression of genes encoding cytokines and chemokines. In addition, IL-1 isoforms are present in psoriatic skin. Oncostatin-M is a potent keratinocyte activator that induces similar effects as TNF-α, IL-1α, IL-17, and IL-22, and regulates many genes related to innate immunity, angiogenesis, adhesion, and motility. The combined effects of all of these selected cytokines appeared to mimic some features of Ps [35]. Recent data has demonstrated that cytokine-treated keratinocyte cells represent a comparable system in which inflammatory markers are upregulated. These markers include antimicrobial peptides, cytokines, and chemokines, as well as the major histocompatibility complex (MHC) molecules.

Co-culture systems have long been used to study the interactions between cell populations and are fundamental to cell–cell interplay research of any kind. Recently, such systems have attracted interest for the study and engineering of complex multicellular synthetic structures. At the basic level, a co-culture is a cell cultivation set-up, in which two or more different populations of cells are grown with some degree of contact between them. The motivations for using such a construct include: (i) studying natural interactions between populations; (ii) creating experimental models; and (iii) generating biomimetic environments of natural systems, such as artificial tissues. Co-cultures are highly relevant for drug research because they provide a more representative human in vivo-like tissue model than animal models and allow for high-throughput testing and in-depth monitoring of drug effects on cell–cell interactions [37]. Before these systems can be managed, they have to undergo rigorous testing for function and safety, especially in terms of the cell–cell interactions and subsequent process optimization [38]. Various attempts to illustrate aspects of Ps using in vitro cell monolayer co-cultures have been reported [39]. Some studies have demonstrated the importance of cross-talk between keratinocyte and lymphocytes or macrophages in modeling the disease in vitro [12,40]. Therefore, for the co-cultivation studies, keratinocyte and lymphocytes or macrophages were grown with an appropriate pore-size barrier placed between them. The cell culture insert was placed on top of the keratinocytes cells to avoid physical contact between the different cell lines. The inflammatory effects on the gene transcription of the proinflammatory cytokine that was induced in the macrophages by treatment with 1 μg/mL lipopolysaccharide (LPS) were analyzed [41,42] (Figure 2b).

### 2.2. Reconstituted Human Epidermal Models (3D)

As already mentioned, cell culture systems have been widely used for biological and pharmacological studies, and some of these have been developed for high-throughput screening of anti-psoriatic drugs. Recent studies have introduced even more advanced systems, such as three-dimensional tissue-engineered human skin equivalents. Some previous investigations have demonstrated the importance of cross-talk between keratinocyte and fibroblast in modeling the disease in vitro [43]. An organotypic culture is a system that accurately models the diseased tissue architecture, with the added potential of the introduction of multiple cell types [44]. Commercially available skin equivalents, mostly derived from foreskin keratinocytes, mimic normal skin and are used in a wide range of biological studies. Various attempts to model aspects of psoriasis (Ps) in vitro have used de-epimerized dermis and adult keratinocytes. The addition of fibroblasts or defined growth factors to the equivalents stimulated the development of epithelium with good morphology. The Chiricozi group has described the genomic response to IL-17 using reconstructed human epidermis (RHE), a full thickness epidermal skin structure consisting of normal human-derived epidermal keratinocytes organized into basal, spinous, granular, and cornified layers, analogously to those found in vivo [45]. Furthermore, RHE was incubated with IL-17, IL-22, or IFN-γ (Figure 2d). All of these models exhibit features of the psoriatic epidermis, and some of them were validated by anti-psoriatic agents [45,46,47,48,49,50].

The Ps 3D tissue model provided by MatTek Corporation (MatTek Corporation, Ashland, MA, USA) closely parallels lesional psoriatic human tissues in terms of morphology and cytokine expression. This model consists of 8–12 cell layers plus stratum corneum (basal, spinous, and granular layers) and exhibits a psoriatic phenotype as evidenced by increased expression of Ps-associated markers, including human β-defensin-2 (HBD2), psoriasin, SKALP/elafin, keratinocyte hyperproliferative cells, and proinflammatory cytokines/chemokines, such as IL-6, IL-8, GM-CSF, and IP-10. MatTek’s Ps tissue model is produced from normal human epidermal keratinocytes, and psoriatic human dermal fibroblasts (PHDF) harvested from psoriatic lesions.

## 3. BioMAP Model

Another approach, named biologically multiplexed activity profiling (BioMAP), provides a characterization of drug function across a broad range of tissue and disease biology. This cellular system characterizes pharmaceutical drug function and is based on statistical analysis of protein expression in a panel of assays using primary human cells stimulated in complex environments. The pattern of activity in the BioMAP system allows the classification of molecules according to the mechanism of action, possibly presenting insights into clinical phenomena [51]. BioMAP profiling has been shown to detect and discriminate multiple functional drug classes, including glucocorticoids, TNF-α antagonists, and inhibitors of calcineurin, HMG-CoA reductase, heat shock protein 90, inosine monophosphate dehydrogenase, phosphodiesterase 4, phosphoinositide kinase-3, and p38 mitogen-activated kinase, among others. The BioMAP system is designed to model complex human disease and tissue biology by stimulating primary human cells (such as endothelial cells, epithelial cells, macrophages, fibroblasts, and keratinocytes), single cell type, or defined mixtures of cell types, such that multiple disease- and tissue-relevant signaling pathways are simultaneously active. The choice of cell type and stimulation is guided by knowledge of certain disease biology and mechanisms. Chemical effects are then recorded by measuring biologically meaningful protein readouts related to significant biological responses (e.g., inflammation, tissue remodeling) [52,53,54].

The BioMAP^®^ Diversity Plus System™ includes 12 specific combinations of human cells stimulated to represent various disease conditions (Figure 2c). This system can also search for similar patterns of biological responses across the BioMAP reference database of >3000 compounds, biologics, and approved drugs and experimental agents. The BioMAP can expose a tissue to a candidate drug and then measure the integrated response, such as tofacitinib or small molecule inhibitor for RORγ/RORγt for the treatment of psoriasis (Ps) [51,55].

## 4. Ex Vivo Models

Ex vivo models are invaluable research tools and have been used to investigate psoriasis (Ps). In general, ex vivo human skin from abdominal surgery is used to study hydrophobic or formulated compounds and is the gold standard for percutaneous absorption studies using Franz diffusion cells. Ex vivo skin is particularly suited to address sun protection with dedicated biomarkers, such as pyrimidine dimers, p53 activation, caspase activation, and sunburn cells, based on histology and immuno-labeling.

One of the commercially available models is provided by Biopta (Available online: www.biopta.com), a company with access to fresh, healthy tissue from operations such as abdominoplasties (Figure 2d). Biopta offers human fresh tissue models using both psoriatic biopsies and normal stimulated skin. As well as a rich source of subcutaneous resistance vessels for vascular studies, this kind of tissue section is also used to provide full thickness biopsies of various sizes. Individual biopsies are then cultured in defined media for some days to provide a biological test system for investigative biology and compound testing. Due to limited treatment options and the need of new therapies treating the underlying causes of Ps, this disease is a major focus of research and development by the pharmaceutical industry; Biopta is involved in the project entitled “Toll-like receptor 7 (TLR7) small molecules in the treatment of cancer and inflammatory disease—Psoriasis”. Biopta will screen these small molecules using fresh human tissue in in vitro assays to select the best candidates. These candidates will then be studied and compared to currently used therapies in well-established humanized animal models of Ps.

## 5. In Vivo Models

Preliminary in vivo research is often used to explain the pathogenic mechanism of illnesses and test the efficacy of new, selected therapeutics [56]. In recent years, some animal models mimicking psoriasis (Ps) have been developed. Except for the HLA-B27 transgenic rat line, current in vivo studies almost always use mouse models of Ps [57,58,59]. Each mice model of Ps reflects a slightly different mechanism of the disease. Thus, each of these models has its strengths and limitations, which are mainly due to the substantial morphological differences between human and mouse skin (except for xenotransplantation models). Unlike mouse skin, the human dermis is thick, and the epidermis is composed of many layers with visible rete ridges. The immune cells found in the human epidermis are mostly Langerhans cells and CD8+ T lymphocytes, whereas the vast majority of murine skin cells are dendritic T cells (DETCs). The dermis of humans and mice is inhabited by mast cells, macrophages, conventional αβ T cells, and innate lymphoid cells (ILCs). Furthermore, many differences are also noticed in early and adaptive immune responses: (1) the activity of leukocytes, defensins, Toll-like receptors, nitric oxide synthases (NOSs); (2) the balance between cytokines, chemokines, and their receptors; (3) the differentiation of Th1/Th2 lymphocytes; and (4) the antigen-presenting function of epithelial cells [56].

The extreme diversity of factors affecting the development of Ps excludes the identification of one principal cause. Therefore, laboratory animals cannot be considered models that precisely reflect the disease. Nevertheless, mouse in vivo models have been used to greatly expand our current knowledge of the causes, pathogenesis, and treatment of Ps [21,60].

The perfect animal in vivo model of Ps should have at least four main characteristics: (1) similar histopathological images; (2) analogous molecular mechanism of disease; (3) increased vascularization; and (4) expected response to drugs commonly applied in Ps [61].

**Figure 3:**
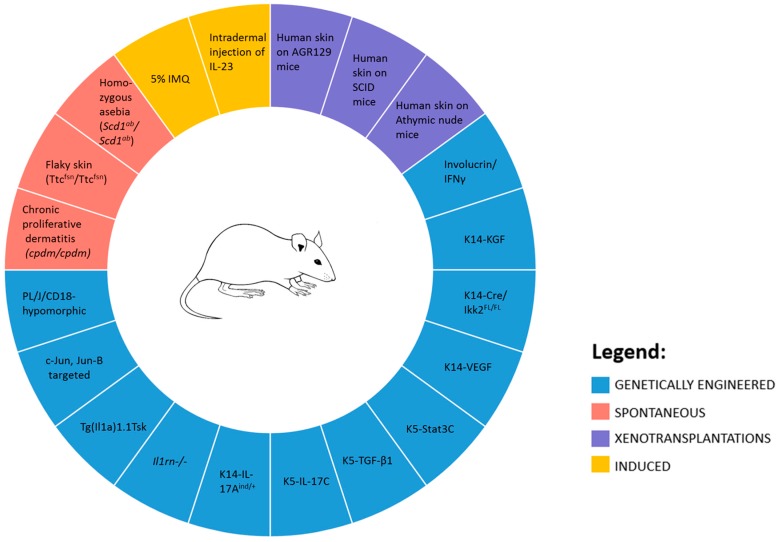
Murine models of Ps.

Murine models of Ps can be divided into spontaneous, genetically engineered (both transgenic and knockout), xenotransplantation, and direct induced [58] (Figure 3).

### 5.1. Spontaneous Mouse Models

There are nearly 100 mouse mutations that result in the appearance of phenotypic features of psoriasis (Ps), such as the thickening of the epidermis or plaque formation. However, due to the lack of T cells influx to the skin, these are mainly used as models to study particular aspects of the disease, such as hyperkeratosis, regulation of neutrophil influence, and angiogenesis [58,62].

Homozygous asebia (*Scd1*^ab^/*Scd1*^ab^) mice were the first in vivo model of hyperkeratosis [63]. The loss of the sebaceous glands is a characteristic feature caused by mutation of the *Scd1* gene (coding the stearyl-CoA desaturase). Thus, the skin of adult *Scd1*^ab^/*Scd1*^ab^ mice becomes scaly, with an epidermal acanthosis, increased vascularization, and an influx of macrophages and mast cells. In the thickened epidermis, long hair follicles run at an acute angle to the deep subcutaneous tissue. The dermis becomes overgrown, with a higher density of fibroblast cells. However, the lack of T cells and neutrophils influence limit the usefulness of this model [60,62].

In contrast, an autosomal recessive mutation in flaky skin mice (*Ttc*^fsn^/*Ttc*^fsn^) leads to induction of inflammation and subsequent hyperkeratosis of stratified squamous epithelia and positive Koebner reaction about two weeks after birth. Also, lymphadenopathy, accumulation of mast cells, increased the influx of neutrophils to the skin, and elevated levels of Epidermal Growth Factor (EGF) receptors and IgE antibodies are observed. However, immunosuppressive treatment with cyclosporine A does not relieve these symptoms. This observation, combined with the limited lifespan of those mice, make this model little in general studies [58,64].

The spontaneous chronic proliferative dermatitis mutation (*Sharpin^cpdm^*/*Sharpin^cpdm^*) shows a variety of Ps features. Mouse skin becomes red and flaky, with expanded blood vessels. Furthermore, the skin becomes inhabited by eosinophils, macrophages, and mast cells. This phenotype can be reversed using corticosteroids, but not by cyclosporine A [62,65].

### 5.2. Genetically Engineered Mouse Models

Progress in molecular biology has enabled the creation of murine models based on the overexpression or the knockout (loss-of-function) of selected genes and their products. Transgenic animals have been developed by targeting specific factors or signaling pathways. Using genetically modified mouse models, it has been possible to clarify the relationship and role of specific cytokines, growth factors, and mediators of the inflammatory response in the process of psoriasis (Ps) pathogenesis [66].

The PL/J/CD18 hypomorphic mouse transgenic model was developed in the early 1990s. These mice carry mutations in the leukocyte β2 integrins. Integrins are responsible for cell–cell contact during various inflammatory reactions. Because of these, the skin of PL/J/CD18 mice is infiltrated by lymphocytes T, resulting in hyperplasia, excessive keratinocytes proliferation with parakeratosis, microabscess formation, and dilation of dermal capillaries. Moreover, the inflammation of PL/J/CD18 hypomorphic mouse skin depends on T cells recruitment, which activates macrophages to release large amounts of TNF-α, one of the main regulators of inflammation [67,68].

Epithelial cells are another target of genetic manipulations. c-Jun and JunB proteins are necessary to control cell differentiation and transformation [69]. Knockout of the factors mentioned above (Tg(Krt1-5-cre/ERT)1Ipc with Junbtm3Wag or Juntm4Wag) in the postnatal mouse skin leads to the appearance of hallmarks common for Ps and arthritis. The skin lesions are infiltrated by neutrophils and lymphocytes. However, other important factors such as IFN-γ, IL-12, and IL-18 were present at only low concentrations or were absent [70].

With the development of our knowledge about Ps, numerous animal models targeting the specific proinflammatory molecules or characteristic signaling pathways have been created. The *Interleukin 1* gene was one of the first to be identified as being expressed in keratinocytes. This cytokine is a key regulator of many processes associated with the immunological response (e.g., activation of proinflammatory cytokines, production of adhesion factors, or enhancing neutrophils, monocytes, and B lymphocytes proliferation). Overexpression of IL-1α in the murine epidermis (Tg(Il1a)1.1Tsk) leads to increased proinflammatory cell infiltration, resulting in hyperproliferation of keratinocytes [71]. Another approach is based on knockout of the IL-1 receptor antagonist *Il1rn*(−/−) in the BALB/c mouse strain. The development of inflammatory response leads to an influx of dendritic cells and T lymphocytes to the skin cells. The epidermis becomes thick, with the presence of parakeratosis and microabscesses. Increased small blood vessel formation in the skin of this mouse model contributes to its resemblance of Ps, which is visible in the histopathological images [72].

The IL-17 cytokine family plays an important role in host response against pathogens, through antimicrobial peptides and proinflammatory cytokine stimulation. Recent studies of Ps have shown that the most elevated types of IL-17 are IL-17A and IL-17C members. K14-IL-17A^ind/+^ mice were created by crossing the IL-17A^ind^ allele to the K14-Cre allele. The inflamed skin of these mice is inhabited by effector T cells, which is confirmed by the presence of T-bet and RORγt, as well as its chemo-attractants: the macrophage inflammatory protein-1α and macrophage inflammatory protein-1β. Another important feature is the appearance of microabscess formed by IL-6R-expressing neutrophils. Due to its immunological similarities to human Ps, this model can be used for a wide spectrum of research [73]. Likewise, histological analysis of K5-IL-17C transgenic mouse skin showed changes such as thickening of the epidermis, hyperplasia, lack of the epidermal granular layer, and increased parakeratosis. Analysis of the expression of the keratinocytes differentiation marker (loricrin) confirmed that those cells remain at an early stage of the development. The K5-IL-17C mouse model exhibits an accelerated angiogenesis, facilitating the influx of T cells (both CD4+ and CD8+), macrophages, and myeloid dendritic cells to the skin. The increased transcript level of proinflammatory factors (such as *IFN-γ*, *IL-1α*, *IL-1β*, *IL-6*, *IL-12*, *IL-17A*, *IL-23*, *S100A8*, *S100A9*, and *TNF-α*) are the essential hallmark of the disease present in the above mouse strain. Moreover, mice treated with the TNF-α inhibitor showed improvement of disease symptoms. Therefore, these transgenic animals seem well-suited for the testing of candidate Ps drugs [74].

IFN-γ is an important factor involved in many inflammatory processes, but mainly in the Th1-mediated immune response. Overexpression of IFN-γ in (CBA × C57/BL10) Fl mice initiates many pathological changes in the skin. Severe symptoms include hair loss, redness of the skin, the presence of flaky lesions, and hair hypopigmentation. The epidermis becomes thickened due to hyperproliferation of keratinocytes. Also, parakeratosis, spongiosis, and the influence of inflammatory cells are present. Thus, the phenotype of these transgenic mice resembles more eczematous than psoriatic changes; nevertheless, this model might be useful for studies addressing the effect of interferon IFN-γ in combination with other proinflammatory factors [62,75].

Another approach concerns the targeting of specific growth factors involved in the development of Ps. The Vascular endothelial growth factor (VEGF) is an essential agent for blood vessels formation. Overexpression of the *VEGF* gene in the mouse epidermis by the K14 promotor contributes to a Ps-like phenotype. The characteristic histological changes seen in this model include parakeratosis, hyperkeratosis, microabscess, and rete ridges in areas of hyperplasia. There is almost no difference between blood vessel development in K14-VEGF mice and humans with Ps. The blood vessels become enlarged, elongated, and tortuous, with the presence of the adhesion molecules (mainly pECAM and *E*-selectin). The elevated levels of T lymphocytes, but also maintaining the ratio of CD4+ cells in the dermis and CD8+ cells in the epidermis, is another significant feature. Importantly, K14-VEGF mice responded to VEGF inhibitor treatment, showing a moderate improvement [76,77]. Similarly, the overexpression of the angiopoietin receptor, Tie2, using the K5 promoter (KC-Tie2 mouse) initiates the process of dermal angiogenesis. Also, overexpression of the *TIE2* gene increased the receptor tyrosine kinase signaling pathways in keratinocytes and led to acanthosis and proliferation of these cells. In addition, genes associated with Ps (e.g., *IFN-γ*, *TNF-α*, *IL-1*, *IL-6*, *IL-12*, *IL-17*, *IL-22*, and *IL-23*) were upregulated [71,78]. A variety of phenotypic features similar to psoriatic skin lesions, which are reversed following treatment with systemic therapeutic, confirmed the usefulness of this model [79]. The transforming growth factor β (TGF-β) family is another group of growth factors involved in Ps development [80]. TGF-β1 mediates differentiation of naive CD4+ T cells to Th17 cells by IL-6-, IL-21-, and IL-23-dependent mechanisms [81,82]. Mice overexpressing TGF-β1 (K5.TGFb1w) develop a severe skin inflammation mimicking human Ps. With the progress of the disease, mouse skin becomes scaly and erythrodermic, with hyperplasia of stratum corneum and acanthosis of the epidermis. Besides, microabscesses appear, consisting of mononuclear cells and neutrophils. Chronic inflammation was supported by angiogenesis and enlarged blood vessels, which increased the inflow of proinflammatory cells and cytokines. In the lesional skin of transgenic mice, genes encoding chemokines (mainly macrophage inflammatory protein [MIP]-1a, MIP-1b, MIP-2, IFN-inducible protein [IP]-10, and monocyte-chemotactic protein [MCP]-1) were upregulated. Moreover, the occurrence of characteristic features, such as Koebner's phenomenon, truly mimics the psoriatic phenotype [78,83].

Keratinocyte growth factor (KGF) belongs to the fibroblast growth factor family and interacts with many epithelial cells, particularly keratinocytes, as a mitogenic intermediary. The KGF receptor (KGFR) plays a crucial role in wound healing and maturation of cells of the epidermal stratum corneum layer [84]. For this reason, creating a K15/KGF mouse model was very promising. Histopathological images of K15/KGF mouse skin resembles the skin changes seen in human Ps. The epidermis becomes thick with overgrowth of the stratum corneum containing immature keratinocytes. However, the development of hair follicles was strongly inhibited. Also, the adipogenesis was suppressed and, therefore, the mice were devoid of fat. The absence of an immune response limits the use of this model in clinical research. However, its application in studies on epidermal aberrations is possible [85].

Beyond targeting the selected factors involved in both the induction and progression of Ps, researchers have developed in vivo models that rely on manipulations of signaling pathways.

Nuclear transcription factor B (NF-κB) is a key player in many inflammatory diseases. Without any specific cellular signals, such as stress or pathogens, NF-κB is blocked by so-called binding inhibitors of κB (IκB). Phosphorylation of IκBb, which is mediated by IκB kinase (IKK), is significant for NF-κB activation [86]. Mice with a deletion of the epidermis-specific nuclear factor κ-B kinase subunit beta (IKK2) gene showed impaired skin homeostasis and, as a result, amplification of severe inflammation. Their epidermis shared many characteristic features of human Ps, including parakeratosis, lack of the granular layer, and microabscess formation. In the dermis, an increased level of T cells, macrophages, mast cells, and granulocytes, together with vascularization, are also observed. Regrettably, increased apoptosis of keratinocytes made this model unsuited to research [87,88].

The signal transducer and activator of transcription protein (STAT) family includes cytoplasmic proteins involved in the transmission of extracellular signals to the nucleus. STATs play an important role in various biological processes, including proliferation, viability, and migration of cells. In Ps, the phosphorylation and excessive activation of STAT3 led to Th17 polarization through an IL-23R-dependent mechanism [89]. In the transgenic K5.Stat3 mouse model, continued expression of the *STAT3* gene in the keratinocytes of the basal layer leads to the appearance of Ps-like skin changes, mainly acanthosis with loss of the granular layer. This process is enhanced by increased blood vessel transformation. There is strong evidence that formation of plaques in K5.Stat3 mice is mediated by T lymphocytes. Intradermal injection of active T cells from STAT3 transgenic mice directly into the graft of immunodeficient mouse skin was able to enhance skin inflammation. The constant expression of the *STAT3* gene can be achieved directly (see above), but also as a result of the mutation of its potential activators. Two the most important factors in this are IL-20 and leptin. However, frequent lack of inflammatory response and incomplete phenotype limit the usefulness of this model [62].

### 5.3. Xenotransplantations Models

An absence of the above-mentioned morphological features of human skin is a major limitation of mouse models of psoriasis (Ps). Xenotransplantations are another approach to develop an animal model of this disease. Xenotransplantations are based on the transplantation of Ps patient’s skin, or its equivalent derived from an in vitro culture, to immune-deficient mice [61]. The athymic nude mouse (Crl:NU(NCr)-Foxn1)^nu^ is a useful model for the study of immunological disorders. Because of its lack of a thymus, and thus the T cells population, the graft (even of tissue obtained from other species) can be maintained without rejection. The first psoriatic xenotransplantation was performed in 1981, initially to clarify the differences between lesional and non-lesional skin [84,90]. Skin taken from a patient was transplanted into nude mice, and the graft was maintained for more than two months, retaining all histological features, including epidermal thickness and papillomatosis. However, certain features of transplanted skin differed from those observed in the human disease, including the retention of the stratum corneum and the lack of parakeratosis. Nevertheless, these studies have shown that the inflammatory reactions seen in the skin tissue strongly affect the disease development [91].

Mice with severe combined immunodeficiency (SCIDs) are widely used as models in Ps research. However, the presence of neutrophils and mature natural killer cells (NKs) are major limitations of these in vivo models. Therefore, single-cell suspension transplants are immediately recognized and lysed by active NK cells. Despite this, the grafts of solid tissue (including psoriatic skin) are not rejected and can be maintained for up to several months. It is inevitable that these grafts undergo changes, such as decreasing in size. Morphology modifications have shown that injecting autologous T cells from a patient directly onto the grafts of SCID mice resulting in a better maintenance of the phenotypic features relative to noninjected controls. This experiment provided evidence for the contribution of T cells to the induction of Ps. This model is still used in pre-clinical research (e.g., for testing new biological agents) [92].

AGR129 mice are deprived of type I and IIIFN receptors and recombinase activating gene-2. This results in a phenotypical lack of B and T lymphocytes; however, in contrast to SCID mice, the NK cells have an abnormal cytotoxic activity and remain inactive, which facilitates the engraftment of human skin. Interestingly, transplantation of patients’ uninvolved skin into AGR129 mice causes the spontaneous formation of plaques, without the need of T cell injection. It has been shown that human T lymphocytes derived from the transplanted skin are activated and proliferated, which induces epidermal lesions. After healthy skin grafting, no psoriatic features were observed, supporting the hypothesis that T cells are an essential factor for the initiation of Ps and play a key role in the pathogenic process [1].

Despite several constraints, because they can mimic almost the entire spectrum of Ps phenotypes, xenotransplantations are one of the best in vivo models of Ps.

### 5.4. Direct Induction

Aldara cream containing 5% imiquimod (IMQ) was initially used as the topical treatment of skin changes caused by human papillomavirus. IMQ was also effective in treating certain forms of cancer, resulting in tumor regression in up to 90% of cases. Interestingly, patients using the Aldara cream reported the appearance of psoriatic lesions, both in the area of use and at distant, unaffected sites. IMQ is a ligand for Toll-like receptors of macrophages, monocytes, and plasmacytoid dendritic cells (pDCs). Therefore, it contributes to a strong activation of the immune system (e.g., enhancing Th1 response or increasing the Langerhans cells migration). These mechanisms have led researchers to develop a targeted, induced mouse model of psoriasis (Ps). BALB/c mice received the drug (5% Aldara) at a dose of 62.5 mg daily for 5–6 days on the back skin. Similar to humans, the daily application of IMQ induced the formation of skin lesions similar to psoriatic plaques. These changes were accompanied by increased epidermal proliferation, erythema, altered vascularity, impaired keratinocytes differentiation (caused by the accumulation of neutrophils in the epidermis), neoangiogenesis, and the influx of CD4+ T cells, CD11c+ dendritic cells, and pDC to the skin. Importantly, it has been shown that IMQ induces epidermal expression of *IL-17A*, *IL-17F*, and *IL-23* genes. Phenotypic changes are partially dependent on the presence of T lymphocytes, and, moreover, the disease does not develop in mice deficient in IL-17 and IL-23 receptors, which is strong evidence of the crucial role of the IL-23/IL-17 axis in Ps [93,94].

IL-23 is composed of p19 and p40 subunits and is produced by antigen-presenting cells. IL-23 is an essential factor for the differentiation of naive lymphocytes to Th17 cells and Th22 cells, which are inherent in the development of skin inflammation [95,96]. Intradermal injection of 1 μg IL-23 into mouse skin stimulates the production of IL-19 and IL-24, which affect keratinocyte differentiation and proliferation through a TNF-dependent mechanism (but not IL-17A). This leads to many histological changes, such as follicular hyperplasia, parakeratosis, and acanthosis. The skin of these mice becomes erythematous with the influx of CD4+ lymphocytes, dendritic cells, neutrophils, and macrophages. Many significant symptoms of Ps are seen in this mouse model, which draws attention to the importance of IL-23 in psoriatic plaque formation [97].

## 6. Conclusions

Prosiasis (Ps) is one of the most common chronic skin diseases. There is currently no drug that can permanently eradicate the skin lesions and completely cure this dermatosis. Therefore, much research is devoted to the discovery of new therapeutic anti-psoriatic substances, which are tested in preclinical laboratory studies and clinical phase trials. Late-stage clinical failure can be largely attributed to a lack of clinical efficacy, indicating a strong need for highly predictive and operative in vivo and in vitro models of Ps. Such models could help in the development of new treatments and, at the same time, translate into significant savings of time and money. Therefore, the pharmacological industry stays alert for the development of even more relevant in vivo and in vitro model systems to improve the success rate of new drugs. The development of a new drug usually takes about two decades. During this period, the pharmaceutical industry spends millions of dollars on the search for new, more effective agents, including the use of multiple psoriatic models. Unfortunately, although many characteristics of the disease are preserved, no model is perfect. Therefore, future research should aim to further optimize existing models, as well as the current strategies for their generation, and aim to identify the exact causes of Ps. Such efforts might lead to a definitive cure for this complex skin disease.

## Figures and Tables

**Table 1:** Model comparisons.

Features	In Vitro Models	In Vivo Models
**Pros**	Easy access to cells Reduced complexity Utilization of human tissue Avoids the need for animals Individual cell responses can be measured	Similar and complex histopathological image Large amount of interacting factors and signaling pathways for manipulation studies or new drugs testing Varied amount of modifications, depending on the purpose of research
**Cons**	Excludes microenvironment influences and might be misleading Excludes macroenvironmental influences	Differences between human and animal skin and immunity Limitation resulting from the polygene nature of the disease—difficulties in a complete phenotype reconstruction by single mutation Influence of environmental factors
**Best use of model**	Evaluation of cell physiology, viability, phenotype, function, and responses to stimulators and inhibitors	Suitable for research the multifarious interactions between skin cells, vascular endothelium and immune response
